# Supplementary material for: Diel and eddy driven changes in microbial gene expression and biogeochemistry in the oceanic chlorophyll maximum
Source: Nat Commun. 2026 Mar 7;17:3636. doi: 10.1038/s41467-026-70228-2 (PMC13096325; doi:10.1038/s41467-026-70228-2)
Supplement: Supplementary file 1 — Supplementary Information [file 41467_2026_70228_MOESM1_ESM.pdf]

Diel and eddy driven changes in microbial gene expression and biogeochemistry in the oceanic chlorophyll maximum

Supplemental Information

Logan M. Peoples, John M. Eppley, Benedetto Barone, Brett W. Hobson, David M. Karl, Brian Kieft, Roman Marin III, Christina M. Preston, Anna E. Romano, John P. Ryan, Christopher A. Scholin, Samuel T. Wilson, Yanwu Zhang, Matthew J. Church, Edward F. DeLong

Spatial variability within the eddy

Simultaneous deployments of two drifters, one focused on the surface and one at 125 m depth, showed that the AUV tracked the subsurface drifter and therefore successfully followed a subsurface water mass (**Supplemental Figure 3**). AUV and CTD sampling locations occurred at an average SLA of -14.9 cm (range, -18.2 to -12.4 cm) across both legs, indicating sampling was firmly within the eddy (**Supplemental Figure 4**). Subsurface Lagrangian sampling equipment all revealed similar horizontal displacement of subsurface water masses away from the eddy center (as reported by the Mesoscale Eddy Trajectory Atlas), with the AUV distance increasing from 21 km during the 1st leg to 33 km during the 2nd. Similar findings were observed for the sediment traps. The shallower sediment trap (100-200 m sampling depths) was deployed near the other Lagrangian equipment and was recovered near the eddy center. In contrast the deeper sediment trap (150-500 m sampling depths), which was deployed a few days later near the edge of the eddy, drifted a considerable distance to the West and likely exited the coherent core of the eddy, resulting in differences in export flux between the two traps. Overall, these findings indicate decoupled movement of subsurface isopycnals relative to surface waters. Therefore, the observed changes in water column properties and biological patterns are the result of both eddy weakening and physical displacement of subsurface water masses away from those at the surface thought to approximate the eddy center. These differing movements of water masses at different depths highlight the complex nature of tracking eddies and the response of microorganisms within these systems.

Overall metatranscriptome expression

The dataset consists of expression patterns of over 4 million unique transcripts across 97 samples. The majority of these transcripts were rare, with approximately 3.8 million transcripts expressed in five or fewer samples. ~130,000 transcripts were present in at least 25 samples, and less than 46,000 were present in 50 or more samples. The top 100,000 most highly expressed transcripts reflected 71% of the total transcript expression, while the top 300,000 reflected ~80%. Total transcript expression ranged between 6.3×10^{10} and 1.7×10^{11} transcripts L^{-1}. Overall, these findings show that the dataset was made up of a few highly expressed transcripts and a large number of rare transcripts.

Comparisons between AUV and CTD sampling

We explored potential differences in metatranscriptome composition depending on whether sampling was performed using AUV *Aku* or traditional CTD casts. The CTD sampling depths were not consistently higher or lower than the AUV during sampling and were typically within 10 m of one another (**Supplemental Figure 12**). Overall transcript expression (i.e., types of

organisms involved) and the total number of unique transcripts expressed per sample was similar between sampling method and across cruise legs. However, total summed transcript abundances were significantly different depending on collection method, with CTD samples showing higher overall transcript abundances than AUV samples ($p < 2e^{-7}$). While this was especially true for the 1st leg ($p < 1.5e^{-8}$), it was also true for the 2nd leg (2nd leg, $p < .015$). NMDS ordinations based on Bray-Curtis dissimilarity showed separation of samples depending on collection method (**Supplemental Figure 12**; PERMANOVA: cruise leg, $R^2 = 0.14$, $F = 17.34$, $p < 0.001$; sampling method, $R^2 = 0.10$, $F = 12.81$, $p < 0.001$). When comparing AUV and CTD samples within their respective legs, ~22,000 and 18,000 transcripts were differentially expressed between sampling methodologies: however, only 3,700 of these transcripts were shared as differentially expressed across both legs. No clear taxonomic patterns were evident amongst these shared transcripts that suggest certain taxa are selected for by method. When AUV samples were compared against CTD samples across both legs simultaneously, 5,600 transcripts were differentially expressed. The total expression of transcripts enriched in the CTD samples was ~3x higher than those enriched in the AUV. Again, differential expression between sampling methods did not appear to be biased towards any specific taxonomic group.

Transcriptomic differences across samples collected by AUV versus CTD rosette could be due to both sampling methodologies and small-scale spatial heterogeneity within the eddy. From the time samples are collected, brought shipboard, and processed, traditional CTD sampling takes longer (2 hrs +) than AUV sampling (~1.5 hrs). CTD rosette-collected communities are exposed to atypical conditions when brought shipboard, causing perturbations in light and temperature that may vary cell physiology. Hence, the filtration time during AUV sampling could induce transcriptional changes, although changes likely also occur during shipboard sampling. The AUV samples were preserved with RNA later immediately following filtration but were retained at temperatures of the chlorophyll maximum for multiple days. This delay in temperature preservation may impact RNA integrity, although both previous estimates (1) and those as part of this study (2) suggest no loss of RNA integrity.

Differences may also be due to spatial variations within the eddy. Conventional CTD sampling during the 1st leg followed the surface drifter and deviated from the location of subsurface Lagrangian instrumentation. In contrast, shipboard CTD sampling generally followed the subsurface Lagrangian instrumentation during the 2nd leg and more closely mirrored the position of the AUV. Regardless, our findings suggest that overall transcript expression patterns were largely similar across AUV and CTD samples. Because AUV and CTD metatranscriptomes generally showed similar expression patterns of major taxa (**Supplemental Figure 6**), and because AUV samples were collected at higher temporal resolution, we largely focus on the AUV samples throughout the main text of the manuscript.

Diel oscillating transcript diversity

We identified transcripts in samples collected by AUV and CTD which showed evidence of diel oscillation across a 24 hr cycle. AUV samples were collected every 3 hours while CTD samples were collected every 6 hours. Daylight (day, 7-18 hrs vs night, 18-7 hrs) was not a statistically significant predictor of total transcript abundance in the AUV (Kruskal-Wallis test, $p < 0.69$) or CTD ($p < 0.5$) samples. We identified 13,735 oscillating transcripts in the AUV samples and 4,118 in the CTD samples. 2,469 transcripts were shared between both sampling methods,

indicating most (60%) of the CTD transcripts also oscillated in the AUV samples but not the other way around. Because the AUV samples were collected at higher temporal resolution, we report the AUV transcripts identified as showing diel oscillation patterns throughout the main text of the manuscript and the figures.

When looking at the diversity of transcripts which showed diel rhythmicity in either AUV leg (**Figure 3**), in excess of 37% of transcripts were related to the Cyanobacteria, 2.8% were related to *Pelagibacter* or *Pelagibacter*_A (391 total transcripts), 0.3% were related to *Nitrosopelagicus* (44 transcripts; including unidentified Thaumarchaeota, 54 transcripts), and 0.3% were related to the genus MGIIb-O3 (38 total transcripts). Nearly 20% of the oscillating transcripts belonged to eukaryotes, with the majority belonging to *Pelagomonas* (1531 transcripts, 11% of total), *Bathycoccus* (302 transcripts, 2% of total), and *Ostreococcus* (206, 1.5% of total). Transcripts which showed diel expression during both legs (3449 total transcripts) also overwhelming belonged to the Cyanobacteria and eukaryotes, represented by *Prochlorococcus*_B (1149 transcripts) and *Pelagomonas* (362 transcripts). The genus CACIJG01 within the Alphaproteobacteria represented 0.5% (16 total transcripts) of the total transcript diversity which oscillated in both legs, the highest represented non-photosynthetic group, highlighting the apparent low overall diel transcript expression within putatively heterotrophic lineages.

Diel oscillating transcript function

The KOs with the highest number of oscillating transcripts included: RuBisCO (*rbcL*, K01601; *rbcS*, K01602), *groEL* (K04077; mostly within members of the Cyanobacteria and Alphaproteobacteria including GCA-002684695, AG-337-I02, and other unidentified members of the Rhodobacteraceae), dynein axonemal heavy chain involved in motility within the eukaryotes (K10408), proteins involved in photosynthesis (i.e., PSII: K02703, K02704, K02705, K02706, K02716; PSI: K02689, K02694; others: K08922, K03403, K02634), RNA polymerase and associated sigma factors (K03043 and K03046 within the Cyanobacteria and eukaryotes), ATP synthase (K02108, K02109, K02111, K02112, and K02116 in the Cyanobacteria and eukaryotes), *dnaK* (K04043), elongation factor tu (K02358) in the Cyanobacteria, eukaryotes, and *Pelagibacter*, *ftsH* (K03798) in the Cyanobacteria and members of the Alphaproteobacteria, including *Pelagibacter*, and N cycling genes including the ammonium transporter (K03320) and glutamine synthetase (K01915).

Microorganisms in the surface ocean may partition their metabolism to certain times of the day, resulting in asynchronous timing of transcript expression between lineages (3). This would allow taxa to mitigate competition for the uptake of limiting resources, such as nitrogen and phosphorus, for example between members of the Cyanobacteria and members of the Alphaproteobacteria. In our dataset we observed some evidence for this behavior. Transcripts for the expression of ammonium transporters (*amt*, K03320) and N-cycle controlling glutamine synthetase (K01915) had a high number of oscillating transcripts and included members of the Cyanobacteria, *Pelagibacter*, and other Alphaproteobacteria, consistent with previous studies (3). In the Cyanobacteria, ammonium transporters were most highly expressed in the afternoon. Within *Prochlorococcus*_B specifically, 39 transcripts were identified as *amtB* (K03320), 14 of which oscillated. These 14 oscillating transcripts reflected 95% of the total transcript expression of *amtB* by *Prochlorococcus*_B, highlighting the significance of the daily temporal cycle on the uptake of ammonium in this genus. These transcripts were consistently expressed in the

afternoon and evening. Ten *amtB* transcripts showed diel expression in *Pelagibacter*. Over 1,000 transcripts in total were putatively identified as *amtB* belonging to *Pelagibacter*. Of these transcripts, the top 2 most highly expressed transcripts oscillate: however, they represent only 15% of the total expression of *amtB* by *Pelagibacter*. Similar findings are evident for glutamine synthetase, which controls nitrogen metabolism. *Prochlorococcus*_B expressed 52 total transcripts identified as *glnA*, with 13 of them oscillating and showing highest expression in the late afternoon. These oscillating transcripts reflected 94% of the total *glnA* expression in *Prochlorococcus*_B. In *Pelagibacter*, four *glnA* transcripts oscillated but represented only 12% of the total expression of *glnA* by *Pelagibacter*. While transcripts involved in nitrate uptake did not show diel rhythms in the cyanobacteria, we did observe diel expression of a nitrate transporter (K02575) in *Pelagomonas* in the morning and an *amtB* transporter at night. These timings are different than the Cyanobacteria and may suggest temporal differences in nitrogen uptake by photosynthetic organisms at the DCM. Overall, while our findings somewhat support previous observations in the surface ocean suggesting that nutrient uptake may be partitioned at different times of the day by different organisms, given the high diversity and inconsistent expression patterns in heterotrophs the timing and importance of this process at the DCM remains unclear in non-photosynthetic lineages.

Because of the importance of diel transcript expression in photosynthesis, we also explored whether light controlled the expression of rhodopsins. We identified over 760 transcripts as putative rhodopsins based on KO categories (**Supplemental Figure 8**), with over 330 belonging to *Pelagibacter*. Of these, only 14 showed evidence of diel expression: 6 of these belonged to *Pelagibacter*. Once again, these findings highlight that while some rhodopsins are clearly controlled across a diel cycle in photoheterotrophic lineages, they reflect a small proportion of the total diversity of expressed rhodopsin transcripts.

Figure 3G shows the expression patterns of transcripts related to the Cyanobacteria within certain functional groups. The Photosynthesis, Carbon fixation, Oxidative phosphorylation, Ribosome, and Nucleotide metabolism (Purine and Pyrimidine metabolism) groupings reflect transcripts identified as belonging to those pathways in the KEGG database, regardless of whether they oscillate or not. The ribosome grouping also included elongation factor tu (K02358), elongation initiation factor IF-2 (K02519), elongation factor ts (K02357), and elongation factor G (K02355). The cell division and circadian clock grouping reflects transcripts functionally identified as *ftsZ* (K03531), *sepF* (K09772), DNA gyrases (K02469, K02470), *kaiC* (K08482), *dnaK* (K04043), *grpE* (K03687), groEL (K04077), groES (K04078), and recA (K03553). The Nitrogen metabolism (N) grouping reflects the transcripts involved in the uptake and incorporation of ammonium, nitrate, urea, nitrate, and nitrite, along with those involved in nitrogen regulation, including glutamine synthetase and glutamate synthase. The superoxide dismutase grouping reflects transcripts identified as sodN (K00518). The RNA grouping reflects RNA polymerase (K03040, K03043, K03046, K03086, K00962). Only transcripts present within at least 10 samples are shown.

Iron limitation at the DCM

Iron concentrations may be an important control on the types of photosynthetic organisms and their metabolism at the DCM, including during eddies (4, 5, 6, 7, 8). We observed transcriptional differences between cruise legs that may reflect changes in iron limitation with eddy strength,

including expression patterns of iron uptake and photosynthetic transcripts (see also main text). We further explored this idea in the Cyanobacteria, a group whose iron-limitation responses are relatively well characterized. Cyanobacteria in culture can alter their metabolism to cope with iron limitation, including by replacing enzymes that require iron with those that do not. For example, cultures of *Prochlorococcus* preferentially replace the photosynthetic enzymes *petF* (iron-containing) with *isiB* (8, 9). The overall ratio of photosystem II:photosystem I enzymes can also be reflective of iron stress, as PSI requires more iron (10). In this study, many iron-related transcripts showed evidence of diel expression, consistent with their function in photosynthesis (**Supplemental Figure 13**). Notably, we observed significantly higher expression of putative iron porin transcripts (based on NCBI annotations) related to *Prochlorococcus*_B during the 1st leg. Ratios of *isiB*:*petF* and *psbA*:*psaC* (representing PSII:PSI), along with the overall expression of iron deficiency (*afuA* or *idiA*) and ferretin transcripts, were generally more highly expressed during the 1st leg (Leg 1:Leg 2 ratio > 1). Ribosomal protein transcripts were used to evaluate whether these differences were due to overall higher expression of the cyanobacteria in one leg vs the other, rather than by differences in iron-related transcripts *per se*. Ribosomal protein transcript expression was also higher during the 1st leg. When comparing iron transcript expression ratios to ribosomal protein ratios (as represented by the dashed line) mixed results were observed, indicating some of the iron patterns may be due to overall higher transcriptional activity by cyanobacteria during the 1st leg rather than iron transcript changes explicitly. Further examination of the *isib*:*petF* and PSII:PSI ratios based on *Prochlorococcus* GTDB ecotype at the genus level showed clear differences based on clade. Regardless of eddy strength, *Prochlorococcus*_B exhibited lower PSII:PSI ratios (*psbA*:*psaC*, higher iron demand) than the ratio obtained from all Cyanobacteria, perhaps indicating clade-specific differences in photosynthesis that may lead to variations in iron demand between clades. In contrast, there was higher expression of *isiB*:*petF* in *Prochlorococcus*_B relative to all Cyanobacteria regardless of leg, indicating *Prochlorococcus*_B may conserve iron through preferential use of *isiB* relative to other cyanobacteria. Within all cyanobacteria together, the *isiB*:*petF* ratio was higher in the 1st relative to the 2nd leg, consistent with increased use of *petF* and a relaxation of iron stress in the 2nd leg. Overall, these findings provide mixed support for iron limitation at the DCM during the eddy and across legs. It is likely that differences in photosystem ratios and iron demand between different clades of Cyanobacteria may structure their distributions but also complicate their use as general markers of iron stress in mixed communities.

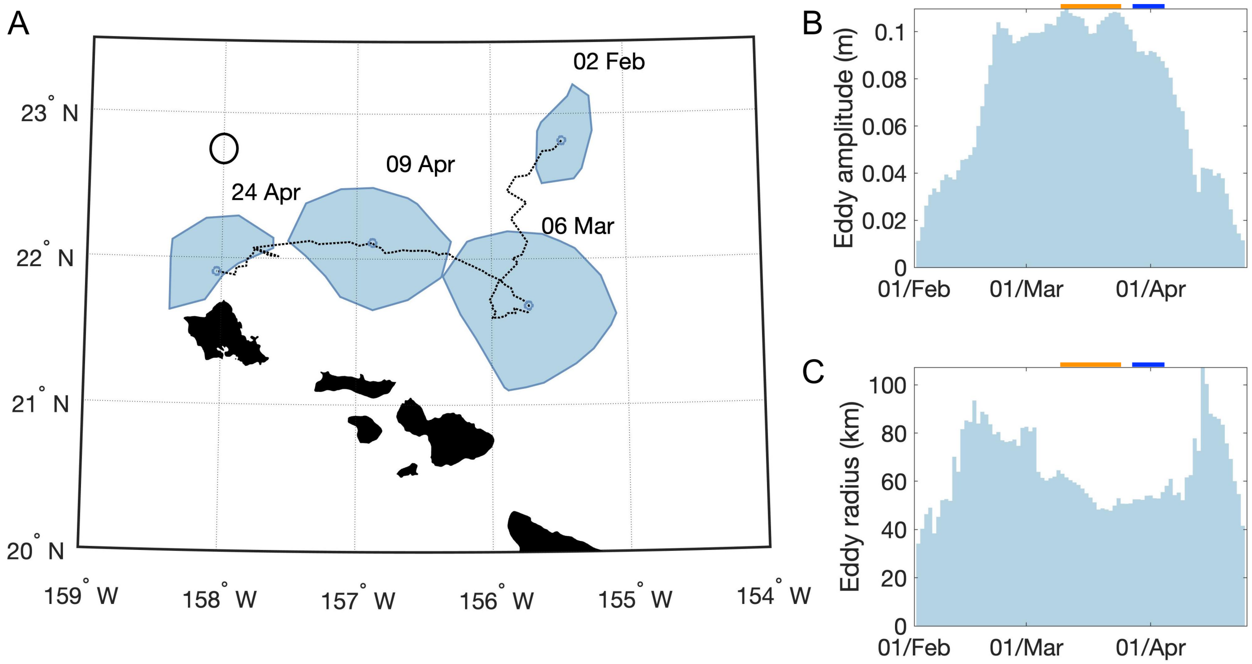

Supplemental Figure 1. Characteristics of cyclone #737324 from the Mesoscale Eddy Trajectory Atlas. The location (**A**), amplitude (**B**), and radius (**C**) of the cyclonic eddy sampled during this study. In A, the dotted line reflects the track of the center of the eddy and the black circle reflects the location of long-term research location Station ALOHA. In **B**+**C**, the orange and blue lines reflect time periods of sampling during Leg 1 and Leg 2, respectively.

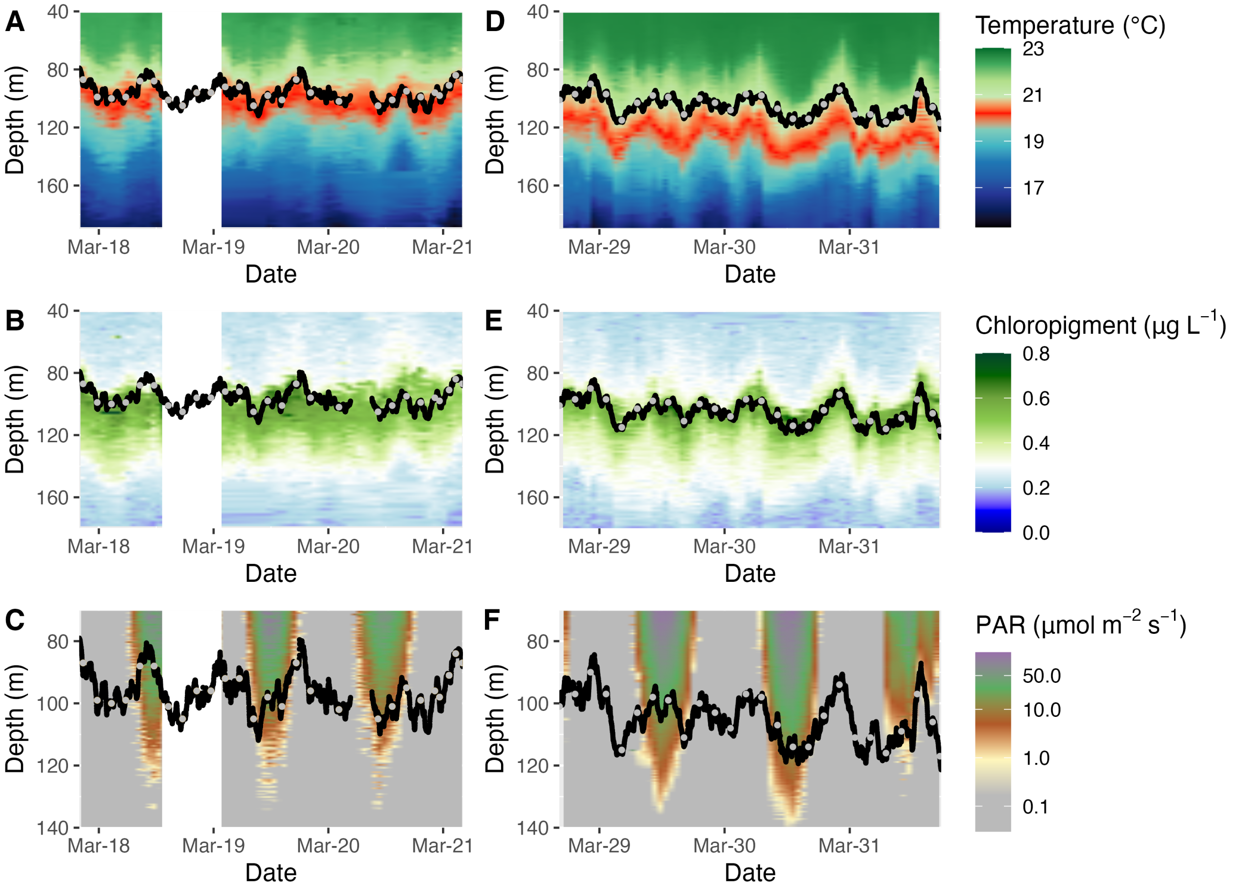

Supplemental Figure 2. Environmental characteristics measured by the AUVs during two continuous three-day periods. Temperature (A+D), fluorescence-derived chloropigment concentrations (B+E), and photosynthetically active radiation (PAR; C+F) measured by AUV *Opah* during the 1st (A-C) and 2nd (D-F) legs of this study. The black line reflects the position of AUV *Aku* at the deep chlorophyll maximum layer and grey dots reflect AUV metatranscriptome sampling locations. Values were interpolated using the R package akima.

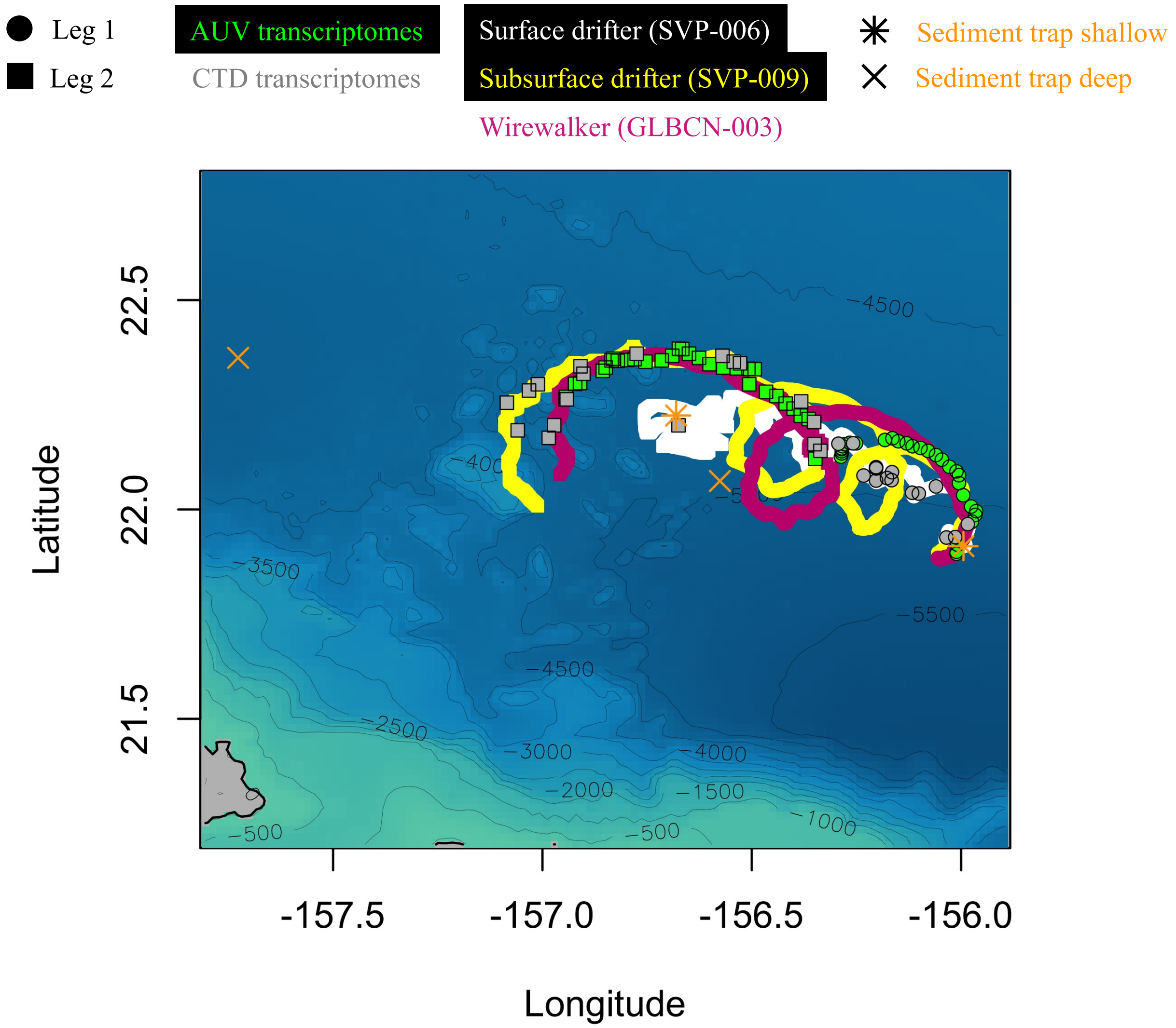

Supplemental Figure 3. Locations of instrumentation deployed during this study. Grey points reflect CTD rosette samples collected by ship. Green points reflect sampling locations of Lagrangian AUV *Aku* at the deep chlorophyll maximum. Purple points reflect the position of a Lagrangian Wirewalker sampling water column properties at depths of up to 400 m. White and yellow points reflect Lagrangian Surface Velocity Program drifter locations with drogues at depths of 15 m and 125 m, respectively. Orange stars and crosses reflect shallow (100-200 m) and deep (150-500 m) sampling sediment traps, respectively. The time of sampling generally moves from right to left across the plot, with deployment legs indicated by shape.

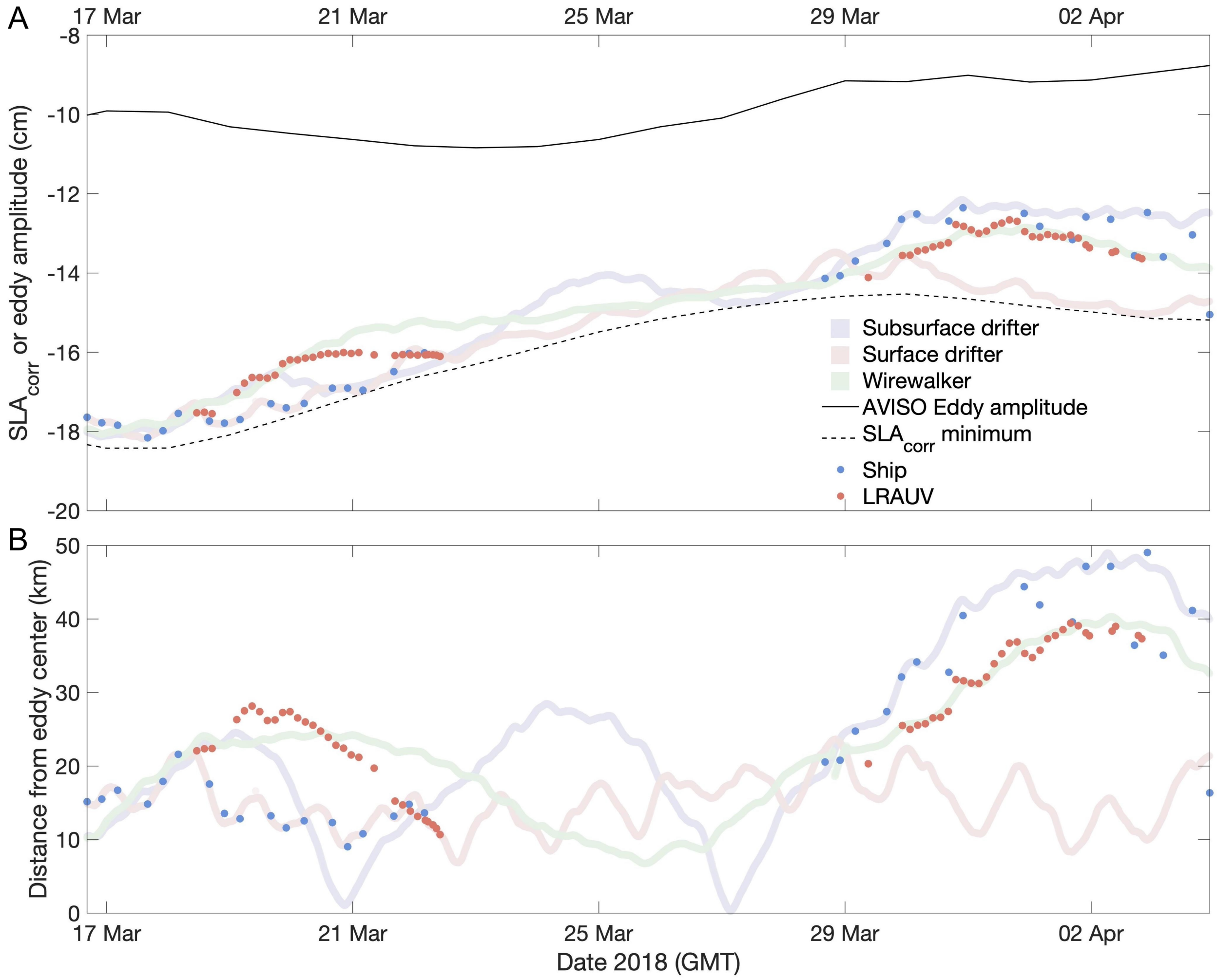

Supplemental Figure 4. Sampling locations relative to eddy conditions and location. Sea level anomaly (SLA_{corr}), eddy amplitude (**A**), and distance from the eddy center (**B**) at sampling locations by the ship and the Lagrangian long range AUVs, Surface Velocity Program drifters, and Wirewalker. SLA_{corr} minimum reflects the minimum value of SLA_{corr} measured in the cyclone.

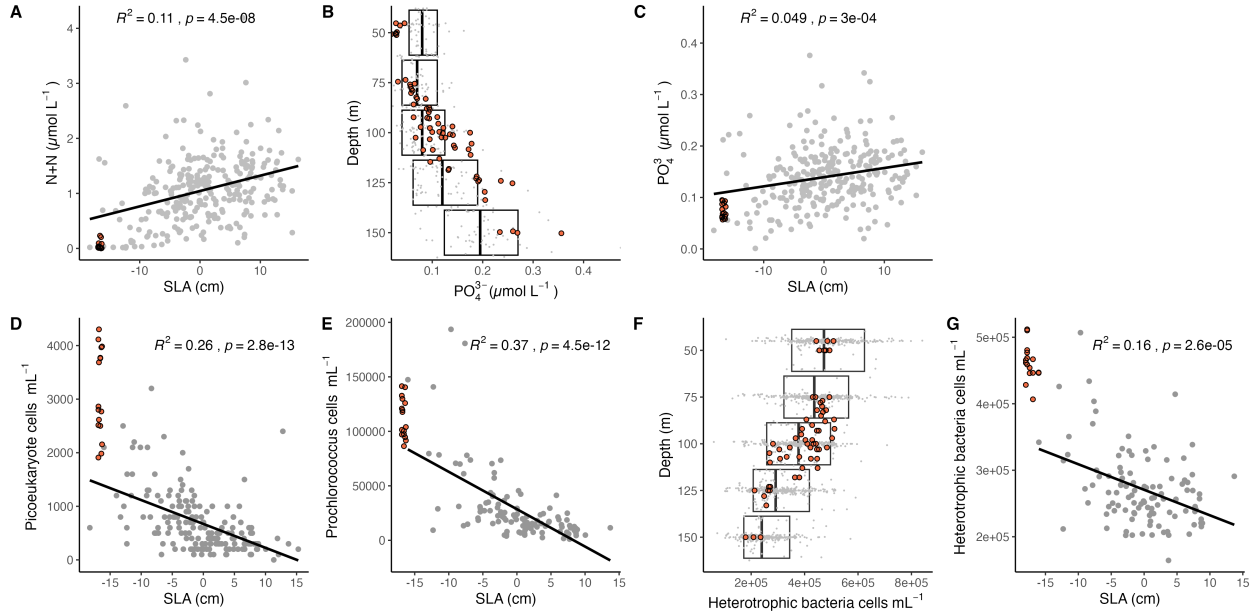

Supplemental Figure 5. Nutrient concentrations and cell abundances relative to typical conditions in the North Pacific Subtropical Gyre. Nitrate+nitrite (N+N; **A**), phosphate (PO_4^{3-}; **B+C**), picoeukaryote abundances (**D**), *Prochlorococcus* abundances (**E**), and non-pigmented heterotrophic picoplankton cell abundances (**F+G**) versus sea level anomaly (SLA) and depth. CTD rosette-collected data in A, C, D, E, and G are from the DCM (this study, orange points, 24.6-24.8 kg m^{-3}) relative to the 24.7 kg m^{-3} isopycnal from the Hawaii Ocean Time-series (HOT) at Station ALOHA (grey points). Lines of best fit and boxplots are fit to the HOT data. Boxplots throughout reflect the median, 25th, and 75th percentiles of data.

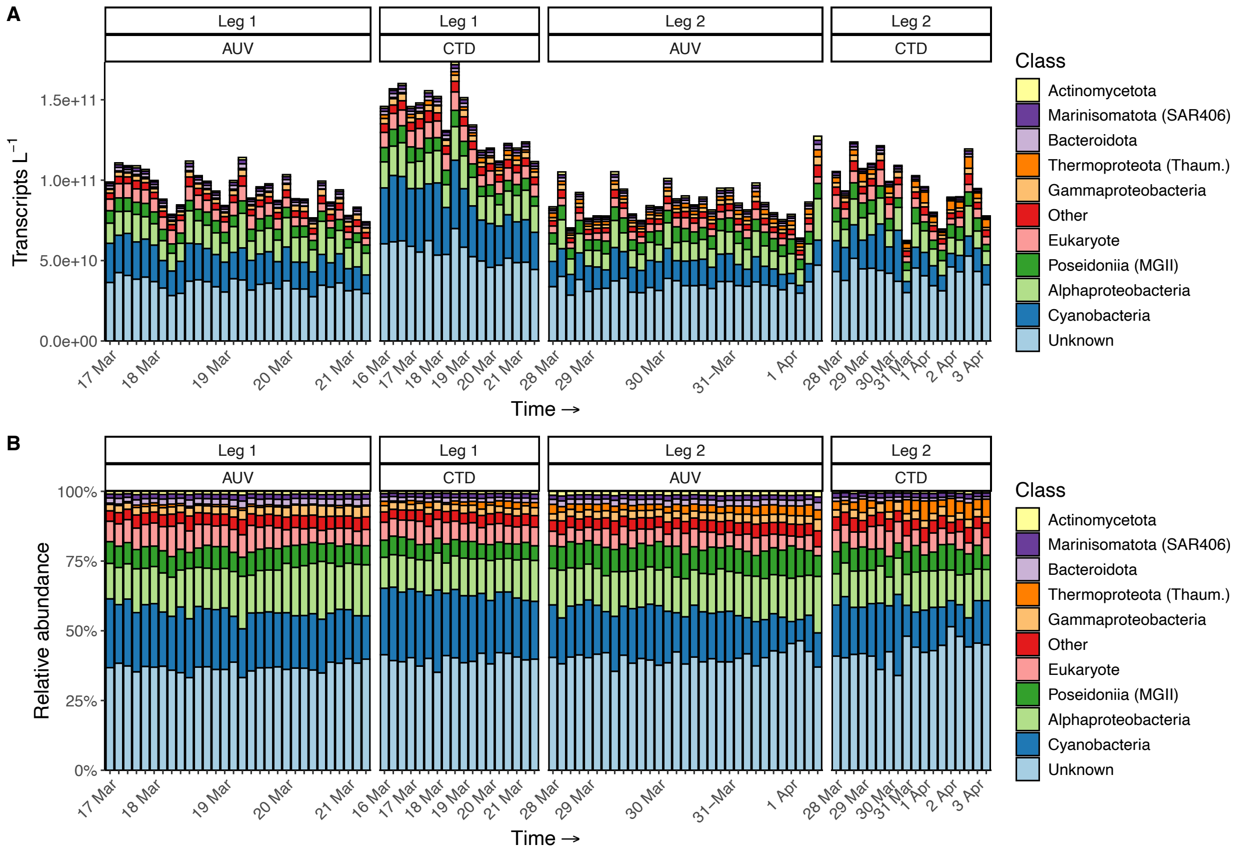

Supplemental Figure 6. Transcript expression by taxonomic group. The total transcripts L^{-1} (**A**) and relative abundances (**B**) per sample of certain taxonomic groups collected by AUV and CTD rosette. Sample names are removed for clarity but are ordered by time of collection from left to right with the first sample collected on each day labeled. Leg 1 samples were obtained when the eddy was stronger, while Leg 2 samples were obtained as the eddy began to weaken.

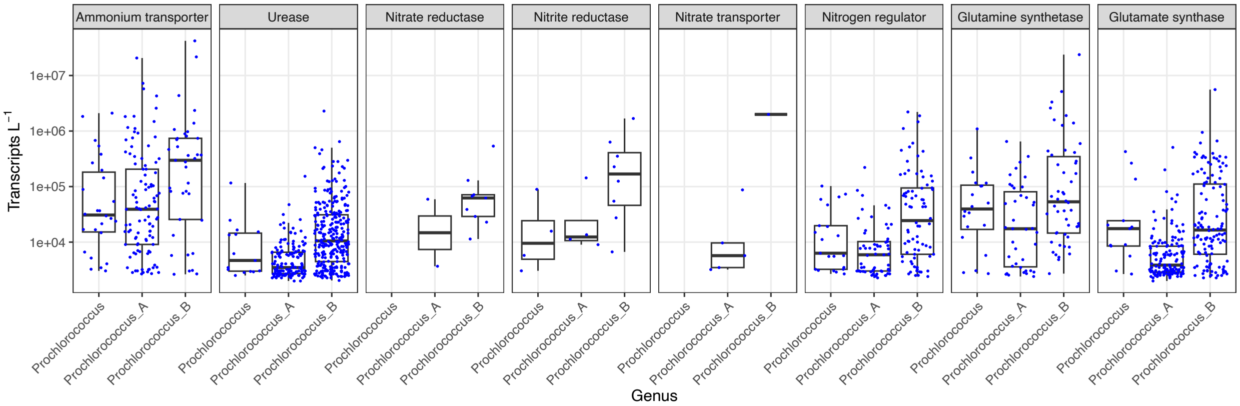

Supplemental Figure 7. The average transcripts L^{-1} per sample of transcripts involved in nitrogen cycling in the cyanobacterial family Cyanobiaceae based on GTDB annotations. Transcripts are grouped by function and included searches for: ammonium transporter (*amtB*, K03320), urease (K01428, K01429, K01430, K03187, K03188, K03189, K03190), nitrate reductase (K00367, K00372, K00360, K10534), nitrite reductase (K00361, K00366, K17877, K26138, K26139), nitrate transporters (K02575, K15579, K22067), nitrogen regulators (K21561, K04751), glutamine synthetase (K01915), and glutamate synthase (K00284). Boxplots throughout reflect the median, 25th, and 75th percentiles of data.

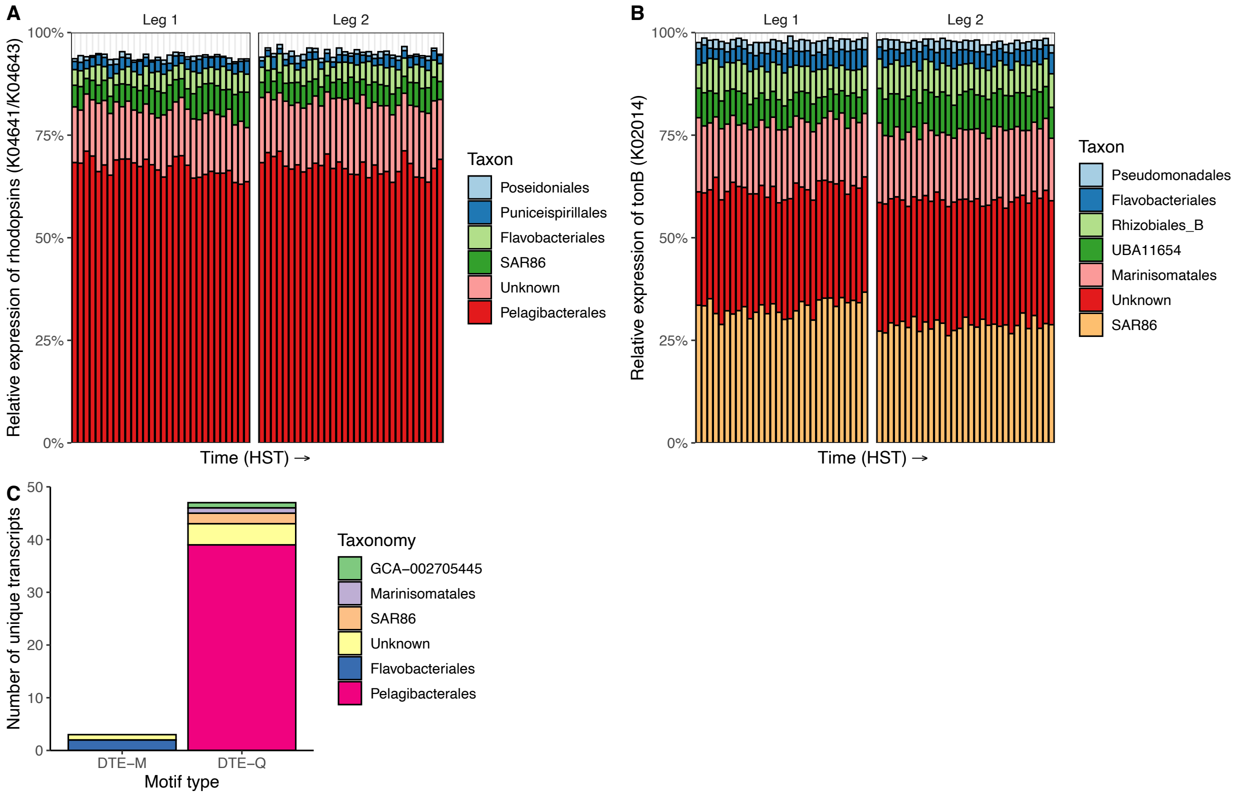

Supplemental Figure 8. The relative expression per AUV sample of rhodopsin (**A**) and tonB-dependent receptor (**B**) KEGG orthologs by taxon. **C**) Amino acid motif analysis of the top 50 most highly expressed rhodopsin transcripts in the dataset. Amino acid motif DTE-Q reflects proton-pumping, blue-light tuned rhodopsins, while motif DTE-M reflects proton-pumping, green-light tuned rhodopsins (see 11, 12).

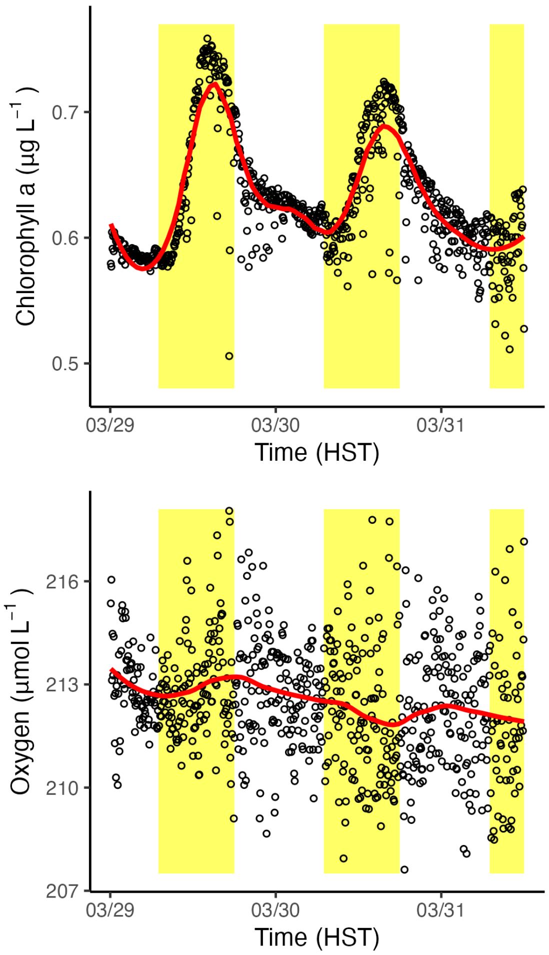

Supplemental Figure 9. Representative chlorophyll a and oxygen (O_2) concentrations measured by AUV *Aku* at the DCM during the second leg. Yellow reflects daylight.

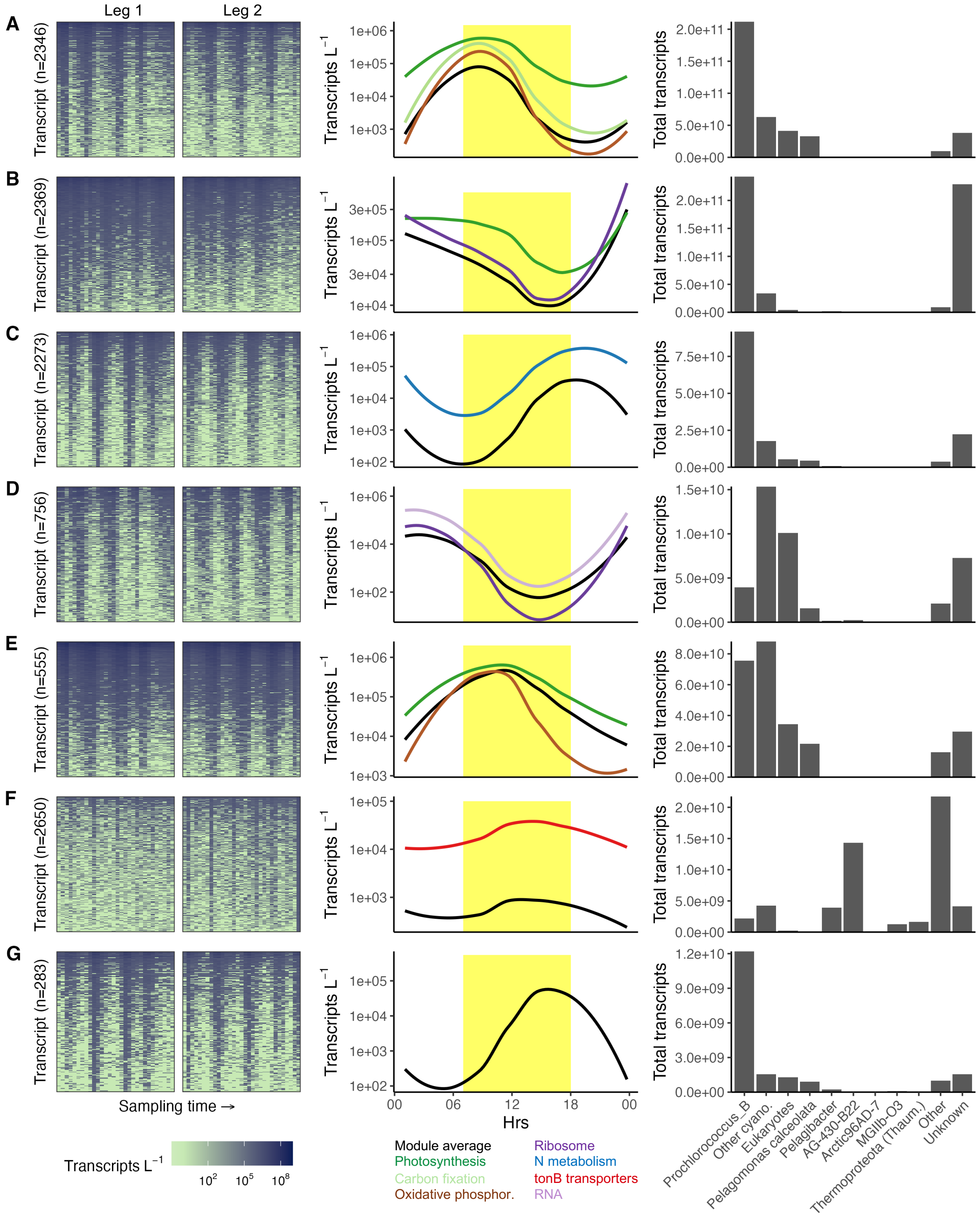

Supplemental Figure 10. Groups (modules, **A-G**) of co-expressed transcripts and their expression patterns, organized from left to right. The left panels show heatmaps of absolute transcript abundances within each group of co-expressed genes. The middle panels show the average expression of all transcripts in that group across a diel cycle (black line) and the average

expression of select KO categories within that group labeled by functional category (colored lines). The right panels show taxon-specific total transcript abundances for co-expressed gene modules across the entire dataset. Groups in A, B, C, D, E, and G show evidence of diel rhythmicity, while F does not.

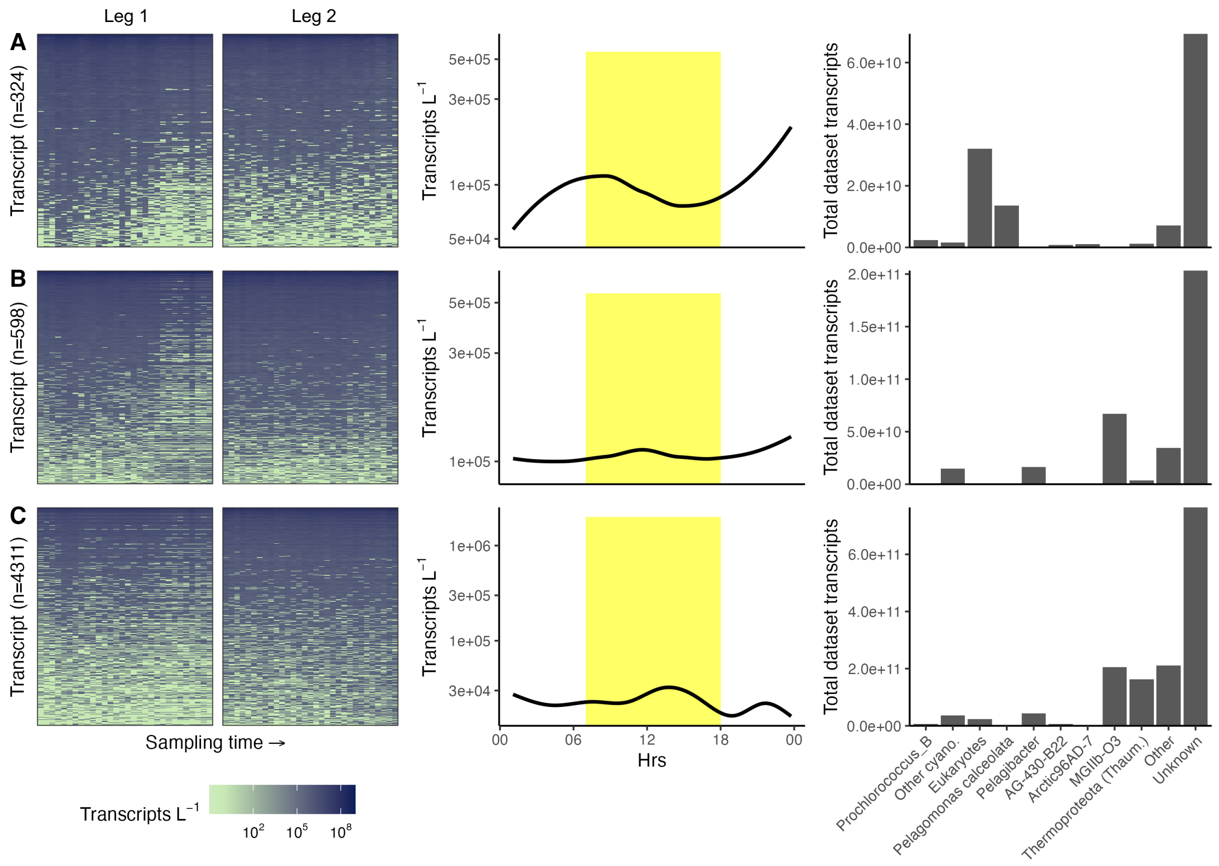

Supplemental Figure 11. Groups of co-expressed transcripts (**A-C**) that show higher expression during one sampling leg relative to the other but do not oscillate across a diel cycle. The left panels show a heatmap of the expression of each transcript within each group. The middle panels show the average expression of all transcripts in that group across a diel cycle (black line). The right panels show the total expression across the dataset of transcripts in that module by taxon.

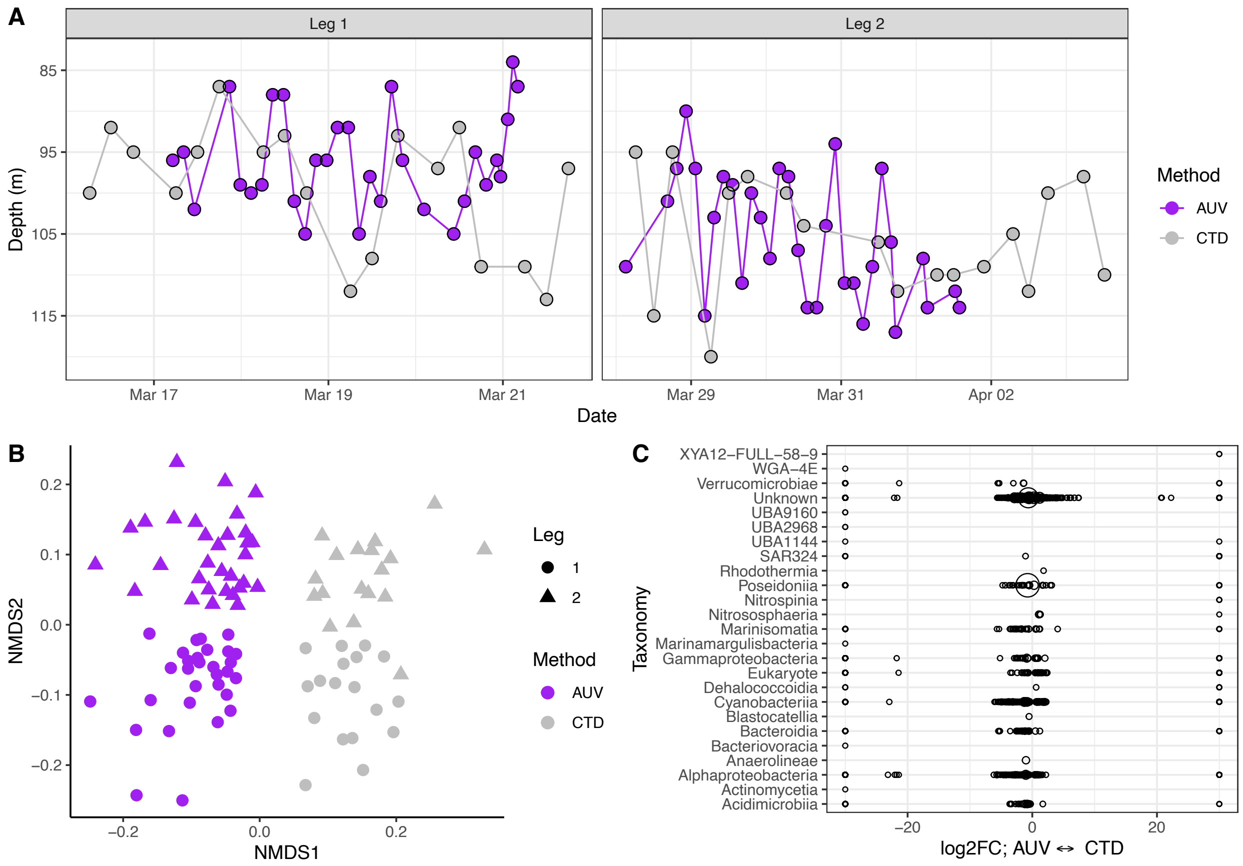

Supplemental Figure 12. Comparisons between AUV and CTD samples. **A**) Sampling depths for transcriptomics at the deep chlorophyll maximum by AUV *Aku* or ship-based CTD rosette. **B**) An NMDS ordination comparing transcript expression across legs and sampling methods (stress = 0.23). The top 300,000 transcripts were used and samples were rarefied to an equal depth. **C**) DESeq2 comparisons identifying transcripts differentially expressed between AUV samples and CTD samples. Comparisons within legs were run separately and transcripts identified as differentially expressed within both legs are shown, with the log2FoldChange (log2FC) reflecting that in the first leg.

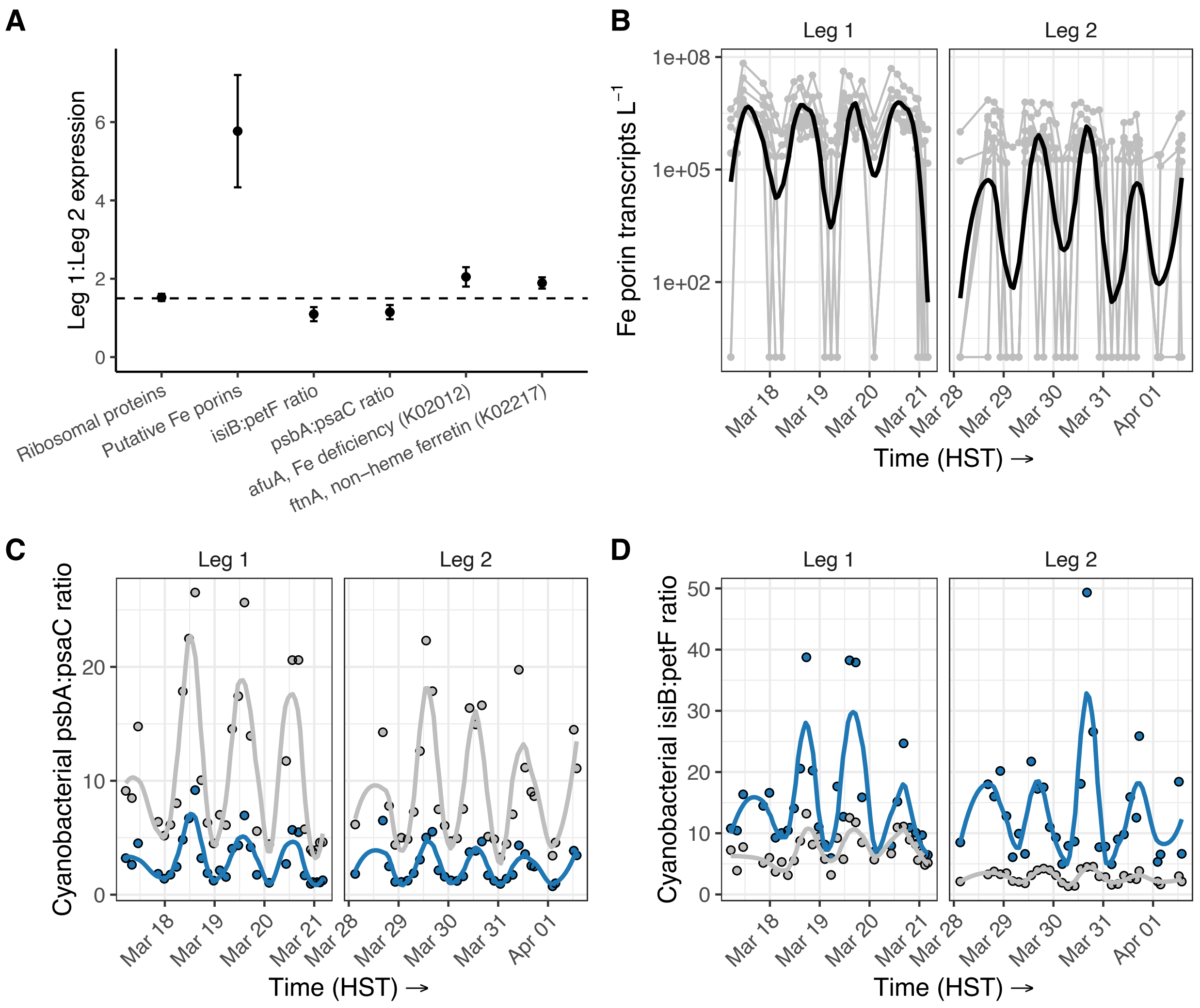

Supplemental Figure 13. Expression patterns of transcripts involved in adaptation to low iron conditions within the Cyanobacteria (as identified using GTDB). **A**) The ratio of transcript expression between the first and second sampling legs. All ratios are > 1. The dotted line reflects the ratio of ribosomal protein transcript expression (see Supplemental Information). **B**) The expression patterns of putative Fe porin transcripts identified in *Prochlorococcus*_B. **C**) The *psbA*:*psaC* ratio, which is a proxy for the PSII:PSI ratio, in all Cyanobacteria (grey) versus just *Prochlorococcus*_B (blue). **D**) The *isiB*:*petF* ratio in all Cyanobacteria (grey) versus just *Prochlorococcus*_B (blue). Error bars reflect standard error.

References

1. Ottesen, E.A., Marin III, R., Preston, C.M., Young, C.R., Ryan, J.P., Scholin, C.A. & DeLong, E.F. Metatranscriptomic analysis of autonomously collected and preserved marine bacterioplankton. *ISME J.* **5**, 1881-1895 (2011). https://doi.org/10.1038/ismej.2011.70

2. Zhang, Y., Ryan, J.P., Hobson, B.W., Kieft, B., Romano, A., Barone, B., *et al*. A system of coordinated autonomous robots for Lagrangian studies of microbes in the oceanic deep chlorophyll maximum. *Science Robotics* **6**, eabb9138 (2021). https://doi.org/10.1126/scirobotics.abb9138

3. Muratore, D., Boysen, A.K., Harke, M.J., Becker, K.W., Casey, J.R., Coesel, S.N., *et al*. Complex marine microbial communities partition metabolism of scarce resources over the diel cycle. *Nat. Ecol. Evol.* **6**, 218-229 (2022). https://doi.org/10.1038/s41559-021-01606-w

4. Doblin, M.A., Petrou, K., Sinutok, S., Seymour, J.R., Messer, L.F., Brown, M.V., *et al*. Nutrient uplift in a cyclonic eddy increases diversity, primary productivity and iron demand of microbial communities relative to a western boundary current. *PeerJ* **4**, e1973 (2016). https://doi.org/10.7717/peerj.1973

5. Hogle, S.L., Dupont, C.L., Hopkinson, B.M., King, A.L., Buck, K.N., Roe, K.L., *et al*. Pervasive iron limitation at subsurface chlorophyll maxima of the California Current. *Proc. Natl. Acad. Sci. USA* **115**, 13300-13305 (2018). https://doi.org/10.1073/pnas.1813192115

6. Ellwood, M.J., Strzepek, R.F., Strutton, P.G., Trull, T.W., Fourquez, M. & Boyd, P.W. Distinct iron cycling in a Southern Ocean eddy. *Nat. Comm.* **11**, 825 (2020). https://doi.org/10.1038/s41467-020-14464-0

7. Browning, T.J., Al-Hashem, A.A., Hopwood, M.J., Engel, A., Belkin, I.M., Wakefield, E.D., Fischer, T. & Achterberg, E.P. Iron regulation of North Atlantic eddy phytoplankton productivity. *Geophys. Res. Lett.* **48**, e2020GL091403 (2021). https://doi.org/10.1029/2020GL091403

8. Hawco, N.J., Barone, B., Church, M.J., Babcock-Adams, L., Repeta, D.J., Wear, E.K., *et al*. Iron depletion in the deep chlorophyll maximum: Mesoscale eddies as natural iron fertilization experiments. *Global Biogeochem. Cycles* **35**, e2021GB007112 (2021). https://doi.org/10.1029/2021GB007112

9. Thompson, A.W., Huang, K., Saito, M.A. & Chisholm, S.W. Transcriptome response of high- and low-light-adapted *Prochlorococcus* strains to changing iron availability. *ISME J.* **5**, 1580-1594 (2011). https://doi.org/10.1038/ismej.2011.49

10. Zhang, X., Sunda, W.G., Hong, H. & Shi, D. Extreme plasticity in the photosystem composition of a low-light *Prochlorococcus* ecotype in response to iron and light. *Limnol. Oceanogr.* **10**, 82-90 (2024). https://doi.org/10.1002/lol2.10441

11. Olson, D.K., Yoshizawa, S., Boeuf, D., Iwasaki, W. & DeLong, E.F. Proteorhodopsin variability and distribution in the North Pacific Subtropical Gyre. *ISME J.* **12**, 1047-1060 (2018). https://doi.org/10.1038/s41396-018-0074-4

12. Yoshizawa, S., Kumaga, Y., Kim, H., Ogura, Y., Hayashi, T., Iwasaki, W., DeLong E.F. & Kogure, K. Functional characterization of flavobacteria rhodopsins reveals a unique class of light- driven chloride pump in bacteria. *Proc. Natl. Acad. Sci. USA* **111**, 6732-6737 (2014). https://doi.org/10.1073/pnas.1403051111
