# Supplementary material for: Diel and eddy driven changes in microbial gene expression and biogeochemistry in the oceanic chlorophyll maximum
Source: Nat Commun. 2026 Mar 7;17:3636. doi: 10.1038/s41467-026-70228-2 (PMC13096325; doi:10.1038/s41467-026-70228-2)
Supplement: Supplementary file 3 — Description of Additional Supplementary Files [file 41467_2026_70228_MOESM3_ESM.pdf]

Description of Additional Supplementary Files

File Name: Supplementary Data 1
Description: Transcriptomic samples used in this study along with their accession numbers.

File Name: Supplementary Data 2
Description: A list of the 200 most highly expressed KO categories in the dataset.

File Name: Supplementary Data 3
Description: Transcripts identified as oscillating in the AUV samples, along with their annotation information.

File Name: Supplementary Code 1
Description: Bioinformatics flags and R code for reproducing main text figures 1-4.
